# Supplementary material for: Promotion of E-Cigarettes on TikTok and Regulatory Considerations
Source: Int J Environ Res Public Health. 2023 May 9;20(10):5761. doi: 10.3390/ijerph20105761 (PMC10217796; doi:10.3390/ijerph20105761)
Supplement: Supplementary file 1 [file ijerph-20-05761-s001.zip › ijerph-2289856-supplementary.pdf]

Promotion of e-cigarettes on TikTok and regulatory considerations

Supplementary Table S1. Codebook used to categorise sampled posts

CODES AND DEFINITIONS	CODING RULE
VIDEO METRICS	
**Likes**	
**Views**	
**Comments**	
**Shares**	
**Followers**	
**Following**	
PRODUCTS	
**E-cigarette or vape device visible**<br>1 = yes<br>2 = no	Are there any e-cigarette or other vape products visible in the post?
**E-liquids or e-juice visible**<br>1 = yes<br>2 = no	Are there any e-juice or e-liquid products visible in the post?
**E-liquid flavour visible**<br>1 = yes<br>2 = no	Does the post indicate a flavour of e-liquid in the post?
**Overt product placement**<br>1 = yes<br>2 = no	How is the vape/tobacco product positioned in the post? Overt (i.e. shown openly, or plainly apparent, and is the main focus of the post)
**Covert product placement**<br>1 = yes<br>2 = no	How is the vape/tobacco product placed in the image? Covert (i.e. not openly displayed, or hidden, and is not the main focus of the image)
**Brand or logo visible**<br>1 = yes<br>2 = no	Is a brand or logo visible or mentioned in the post? (includes in the background and on clothing, equipment, and merchandise)
SENTIMENT	
**Sentiment**<br>1 = positive<br>2 = negative<br>3 = N/A	Positive – in favour of e-cigarettes and vaping related products and use<br>Negative – against e-cigarettes and vaping related products and use
**Pro-vaping for vaping/e-cigarette**<br>1 = yes<br>2 = no	Is the post pro-vaping?
**Anti-vaping/e-cigarette**<br>1 = yes<br>2 = no	Is the post anti-vaping?

**Violates content policy**<br>1 = yes<br>2 = no	Does the post/video violate TikTok content policy for e-cigarettes? * See community guidelines (tiktok.com)
TYPE	
**Product review**<br>1 = yes<br>2 = no	Is the post alerting viewers to a product through a review?
**Promoting vape product for purchase**<br>1 = yes<br>2 = no	Is the post promoting a vape product for purchase?
**Monetary promotional offer**<br>1 = yes<br>2 = no	Is the post providing monetary offers?<br>Monetary (any promotional deal that saves you money, lowers the cost of a purchase or changes the cost of the purchase, e.g. coupons, refunds, rebates, two for one deals, and cents-off promotions)
**Non-monetary promotional offer**<br>1= yes<br>2 = no	Is the post providing non-monetary offers?<br>Non-monetary (any promotional deal that does not lower the cost of a purchase, e.g. contests, giveaways, sweepstakes, free shipping or free gift with purchase)
**Sponsored advertisement**<br>1 = yes<br>2 = no	Does post contain the terms #sponsorship or #ad?<br>On TikTok, you must enable the Branded Content toggle when posting branded content. When enabled, it adds a disclosure (e.g. #ad) to the description of your post
**Business affiliation**<br>1 = yes<br>2 = no	Does the post refer or link to a vaping related retailer/distributor? (outlets and companies that sell or distribute e-cigarettes, online or physical store)
CONTENT	
WARNINGS	
**Nicotine warning visible**<br>1 = yes<br>2 = no	Does the post display or refer to a nicotine warning?
**Addiction**<br>1 = yes<br>2 = no	Does the post reference nicotine or vape addiction?
**Health warning visible**<br>1 = yes<br>2 = no	Does the post display or refer to a health warning?
HEALTH-RELATED CONTENT	
**Positive health effects**<br>1 = yes<br>2 = no	Does the post reference general positive health effects of vaping?
**Negative health effects**<br>1 = yes<br>2 = no	Does the post reference general negative health effects of vaping?

**Quit smoking**<br>1 = yes<br>2 = no	Does the post promote vaping as a means to quit smoking? (include quit and cessation hashtags)
**Healthier than smoking**<br>1 = yes<br>2 = no	Does the post specifically compare vaping to smoking, stating vaping is the healthier alternative/option?
**Public health**<br>1 = yes<br>2 = no	Does the post reference public health professionals, organisations or journals?
**Regulation or policy**<br>1 = yes<br>2 = no	Does the post comment on e-cigarette regulation/policy?
E-CIGARETTE USE CONTENT	
**Safety**<br>1 = yes<br>2 = no	Does the post reference how to use e-cigarettes or handle nicotine, battery, or e-liquid 'safely'?
**Customisation**<br>1 = yes<br>2 = no	Does the post depict or reference modifying or 'building' vape products/accessories or making or mixing juices?
**Malfunction**<br>1 = yes<br>2 = no	Does the post reference e-cigarette devices malfunctioning?
**Vape tricks**<br>1 = yes<br>2 = no	Does the post depict or reference vape tricks, or demonstrate how to do tricks?
VIDEO FEATURES AND CHARACTERISTICS	
**Identity or community**<br>1 = yes<br>2 = no	Does the post convey a vaping social identity or shared community affiliation? (determined by these hashtags in post #vapelife, #vapelyfe, #vapecommunity, #vapeworld, #vapeporn, #ecigmafia, #vapefam, #vapefamily)
**Animation**<br>1 = yes<br>2 = no	Does the post contain animations or cartoons?
**Humour**<br>1 = yes<br>2 = no	Does the post attempt to provoke laughter and provide amusement?
**Music**<br>1 = yes<br>2 = no	Does the post contain background or theme music?
**Video length**<br>1 = 1.5 min and under<br>2 = Over 1.5 min	How long is the length of the post?
